# Supplementary material for: Large-bodied sabre-toothed anchovies reveal unanticipated ecological diversity in early Palaeogene teleosts
Source: R Soc Open Sci. 2020 May 13;7(5):192260. doi: 10.1098/rsos.192260 (PMC7277248; doi:10.1098/rsos.192260)
Supplement: Supplementary Information and Figures for: Large-bodied sabre-toothed anchovies reveal unanticipated ecological diversity in early Palaeogene teleosts [file RSOS192260supp1.docx]

**Supplementary Information for:**

**Large-bodied sabre-toothed anchovies reveal unanticipated ecological diversity in early Palaeogene teleosts**

Alessio Capobianco^1,2^*, Hermione T. Beckett^3,4^, Etienne Steurbaut^5,6^, Philip D. Gingerich^1,2^, Giorgio Carnevale^7^, Matt Friedman^1,2^

^1^Department of Earth and Environmental Sciences, University of Michigan, Ann Arbor, MI, USA

^2^Museum of Paleontology, University of Michigan, Ann Arbor, MI, USA

^3^Department of Earth Sciences, University of Oxford, Oxford, UK

^4^King’s High School for Girls, Warwick, UK

^5^Royal Belgian Institute of Natural Sciences, Brussels, Belgium

^6^KU Leuven, Leuven, Belgium

^7^Dipartimento di Scienze della Terra, Università degli Studi di Torino, Torino, Italy

*e-mail acapo@umich.edu

**Geographic and stratigraphic setting of †*Clupeopsis***

In the late 1800s and early 1900s the area north of Chièvres, about 48 km southwest of Brussels, was renowned for its well-preserved fossil fish skeletons. They all came from the same clay pit, located along the east flank of a small hill, about 1.2 km northeast of the Chièvres town centre (map Blicquy-Ath 38/5-6, coordinates: x = 110.725, y = 143.237; GPS coordinates: 50°35’52.37”N, 3°48’52.35”E). The exploitation of this pit, also known as the Dubois clay pit, ceased in the 1930s. The fish skeletons, of which at least four could be saved and stored in the collections of the Royal Belgian Institute of Natural Sciences of Brussels (RBINS), were collected during two exploration campaigns. The first campaign, directed by Delvaux in the middle of the 1880s, led to the discovery of three fairly complete specimens of a single species, identified as †*Osmeroïdes insignis* by Delvaux & Ortlieb (p. 69, pls 1-2) [1] but subsequently named as †*Halecopsis insignis* (Delvaux & Ortlieb, 1887) by Casier [2]. The second campaign was carried out by the scientific and technical staff of the RBINS in 1921 and 1924. Among the few additional fish specimens that could be retrieved from the clay pit at that time is the type and only specimen of †*Clupeopsis straeleni* Casier, 1946 that is re-examined in this study.

On the basis of their personal observations and information provided by the quarry owner, Delvaux & Ortlieb [1] concluded that the fossil fish skeletons were found in small sandy clay lenses at the top of an olive-green coloured stiff clay, about 3.70 m above the Ypresian basal gravel. In the Chièvres area, this stiff clay, which also includes thin lenses of yellowish sand, is overlain by a 1.1 m thick sandy clay. The latter comprises pyritised lignite with pyritised wood fragments, phosphatic nodules of different dimensions and shapes, fish and crustacean debris, and fine-grained septaria-like calcareous (sideritic?) concretions.

As the lower Ypresian of Belgium is marked by SE-NW trending facies belts (Steurbaut & King, p. 138) [3], the palaeogeographic situation of the Chièvres area can be best compared with that of the Quenast quarry, 25 km east of Chièvres[4]. In this pit the Ypresian succession comprises (in ascending order): a basal gravel bed, overlying microdiorite rocks of Late Ordovician age (433 Ma [5]), stratified sandy clays, a thin compact stiff clay and a second thin gravel bed. This succession is less than 1 m thick. The upper gravel bed may be related to the nodule-rich lignitic bed at Chièvres, which represents a condensed facies, perhaps due to sediment starvation. The latter, which also bears evidence of episodic recurrence of swamp conditions, may be correlated with a wood-rich level in the Kester borehole (28.5 km northeast of Chièvres), approximately 8 m above the base of the Ypresian (Steurbaut, unpublished). As the fish skeletons of Chièvres occur just below the condensed bed, at the top of a heterogeneous clayey-sandy facies, it is logical to assume that they derive from the lowest non-calcareous part of the Orchies Clay Member, at the transition to the underlying Mont-Héribu Member (Steurbaut & King, fig. 2) [3]. The same conclusion can be drawn from the analysis of the matrix surrounding the skeleton of †*Clupeopsis*, which led to the identification of non-calcareous interbedded stiff clays and sandy clays. No useful biostratigraphic information could be retrieved from this sample, being non-calcareous and too small to perform a successful dinoflagellate cyst investigation. Cross-correlation between the lower Ypresian lithostratigraphic succession [6, 7] and the Ypresian carbon isotope curve[8] also reveals that the fish skeletons accumulated in the interval between the base of the Ypresian s.s., dated at approximately 54.40 Ma, and the ETM2, dated at 54.05 Ma (dates from ref. [9]). With the base of the Ypresian s.s. is meant the base of the Ypresian clay facies as originally defined by Dumont [10] in the stratotype area of Belgium (base of the Mont-Héribu Clay Member). According to the information of Leriche [11] and Casier [2] and leaving aside the commonly occurring isolated fish fragments and fish debris at the very base of the Ypresian s.s., we may assume that the fossil fishes from Chièvres are among the oldest Ypresian teleost skeletons from Belgium.

**Individual scanning parameters**

Fossil specimens described in this work and extant comparative specimens were studied using micro-computed tomography (μCT) datasets produced using Nikon XT H 225ST industrial μCT scanners at the University of Michigan and the Natural History Museum, London. Individual scanning parameters of new tomograms generated for this study are given below:

†*Clupeopsis straeleni* MRHNB IG 8630. Voltage: 210 kV; current: 200 μA; exposure: 1000 ms; filter: 1.5 mm tin; reflection target: tungsten; effective pixel size: 65.432 μm; scanning facility: NHM, Imaging and Analysis Centre.

†*Monosmilus chureloides* gen. et sp. nov. UMMP GSP-UM 30. Voltage: 190 kV; current: 205 μA; exposure: 1415 ms; filter: 1.5 mm copper; reflection target: tungsten; effective pixel size: 57.94 μm; scanning facility: University of Michigan CTEES.

*Odaxothrissa mento* UMMZ 195016. Voltage: 95 kV; current: 175 μA; exposure: 1000 ms; filter: none; reflection target: tungsten; effective pixel size: 19.97 μm; scanning facility: University of Michigan CTEES.

*Chirocentrus dorab* UMMZ 238306. Voltage: 105 kV; current: 160 μA; exposure: 1000 ms; filter: none; reflection target: tungsten; effective pixel size: 36.43 μm; scanning facility: University of Michigan CTEES.

*Chirocentrus nudus* UMMZ 213502. Voltage: 105 kV; current: 160 μA; exposure: 1000 ms; filter: none; reflection target: tungsten; effective pixel size: 39.00 μm; scanning facility: University of Michigan CTEES.

*Setipinna* [*Lycothrissa*] *crocodilus* UMMZ 209911. Voltage: 100 kV; current: 170 μA; exposure: 1000 ms; filter: none; reflection target: tungsten; effective pixel size: 22.55 μm; scanning facility: University of Michigan CTEES.

*Lycengraulis grossidens* UMMZ 143053. Voltage: 100 kV; current: 170 μA; exposure: 1000 ms; filter: none; reflection target: tungsten; effective pixel size: 22.55 μm; scanning facility: University of Michigan CTEES.

**Supplementary Figures**


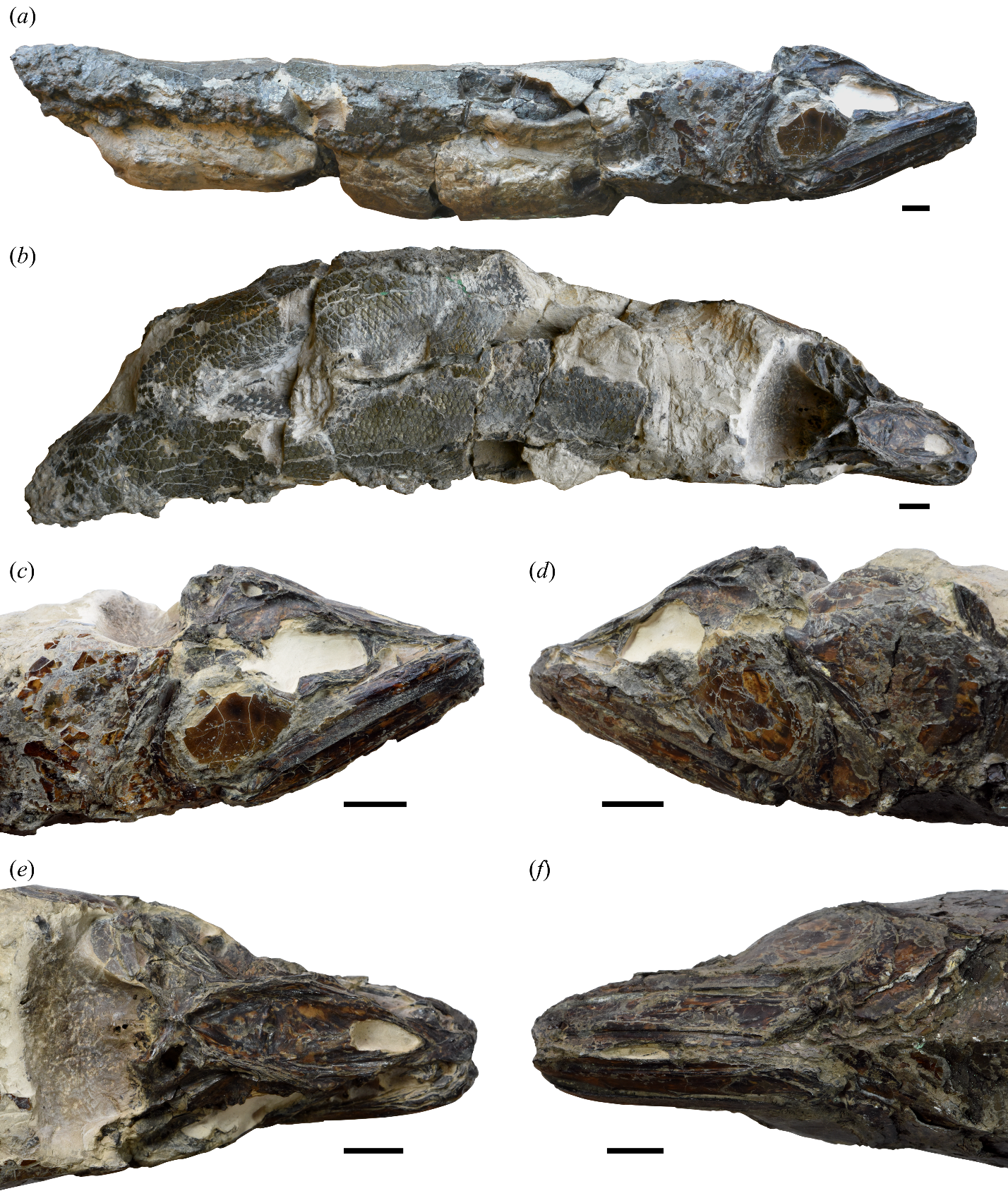


**Supplementary Figure S1.** †*Clupeopsis straeleni* Casier 1946 (MRHNB IG 8630, holotype). Photographs of the holotype in (*a*) right lateral and (*b*) dorsal views. Photographs of the cranial region of the holotype in (*c*) right lateral, (*d*) left lateral, (*e*) dorsal and (*f*) ventral views. Scale bars: 10 mm.

**
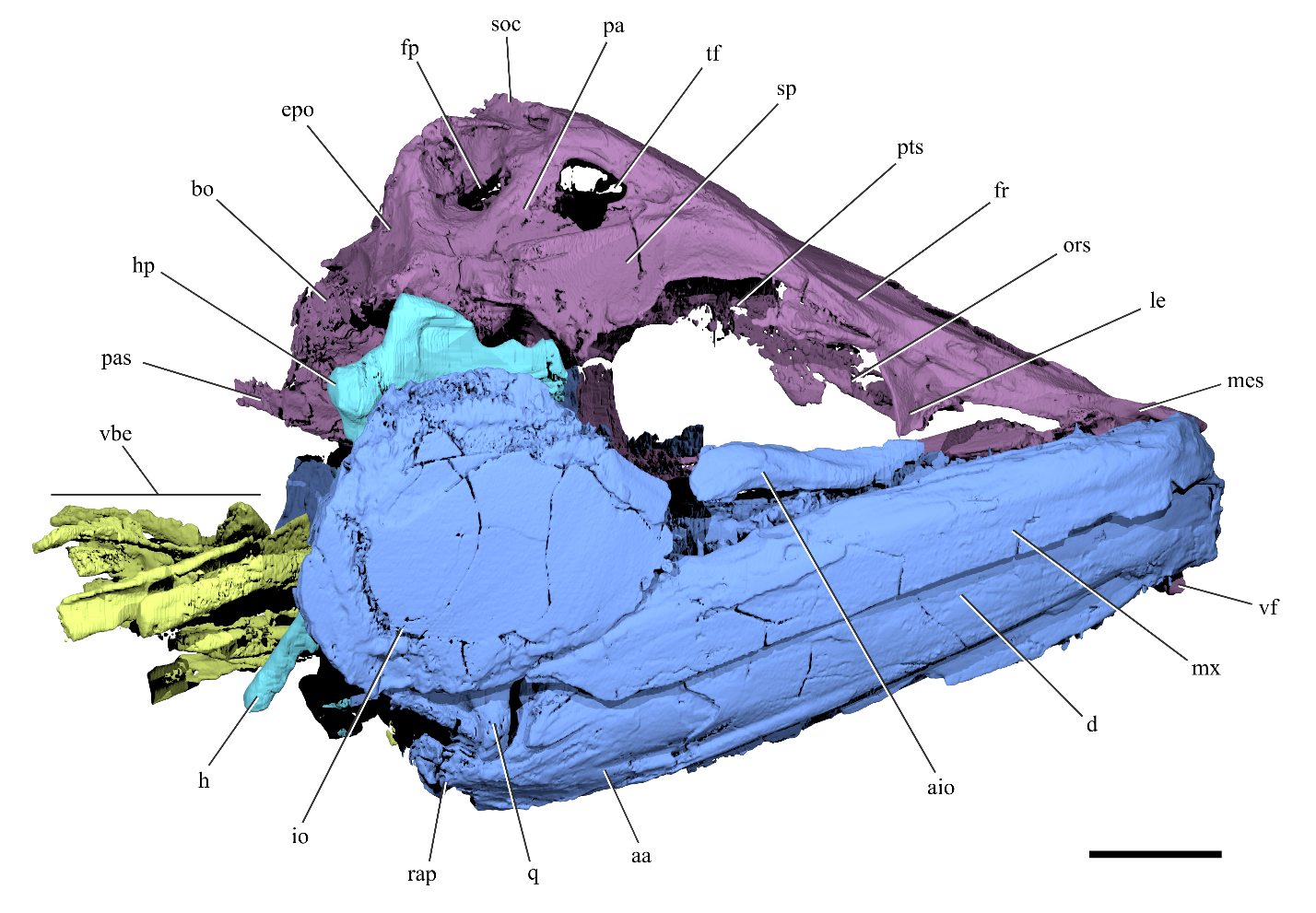
**

**Supplementary Figure S2.** Cranial anatomy of †*Clupeopsis straeleni*. Rendering of †*Clupeopsis* skull in right lateral view, excluding the opercular series. Colors indicate different cranial regions: neurocranium (purple), jaws, palatoquadrate and cheek bones (blue), dorsal portion of hyoid arch (cyan), branchial skeleton (chartreuse). Scale bar: 10 mm. Abbreviations: aa, anguloarticular; aio, anterior infraorbital; bo, basioccipital; d, dentary; epo, epioccipital; fp, pre-epiotic fossa; fr, frontal; h, hyomandibula; hp, hyomandibular process; io, infraorbital; le, lateral ethmoid; mes, mesethmoid; mx, maxilla; ors, orbitosphenoid; pa, parietal; pas, parasphenoid; pts, pterosphenoid; q, quadrate; rap, retroarticular process; soc, supraoccipital; sp, sphenotic; tf, temporal foramen; vbe, ventral branchial elements; vf, vomerine fang.

**(*next page*) Supplementary Figure S3.** Neurocranium of †*Clupeopsis straeleni*. Renderings of †*Clupeopsis* neurocranium in (*a*) dorsal and (*b*) ventral views. Scleral ring and vomerine fang not shown for clarity. (*c*) Rendering of †*Clupeopsis* neurocranium in left lateral view, with partial transparency to reveal the morphology of the *recessus lateralis*. The *recessus lateralis* chamber has been digitally filled to highlight its extent and its external openings in the braincase. Identities of the *recessus lateralis* openings are comparatively based upon ref. [12]. Scale bar: 10 mm. Abbreviations: acr, accessory temporal sensory canal opening to the *recessus lateralis* chamber; afn, anterior frontal fontanelle; aor, anterior opening to the *recessus lateralis* chamber; exo, exoccipital; fp, pre-epiotic fossa; fr, frontal; frco, opening in frontal to supraorbital canal; frlc, longitudinal crest of frontal; le, lateral ethmoid; mes, mesethmoid; mor, middle opening to the *recessus lateralis* chamber; ors, orbitosphenoid; pa, parietal; pas, parasphenoid; por, posterior opening to the *recessus lateralis* chamber; ptg, post-temporal groove; pro, prootic; pto, pterotic; pts, pterosphenoid; rec, *recessus lateralis* chamber; soc, supraoccipital; sp, sphenotic; sr, scleral ring; tf, temporal foramen; vf, vomerine fang; vo, vomer; vots, vomerine tooth socket.


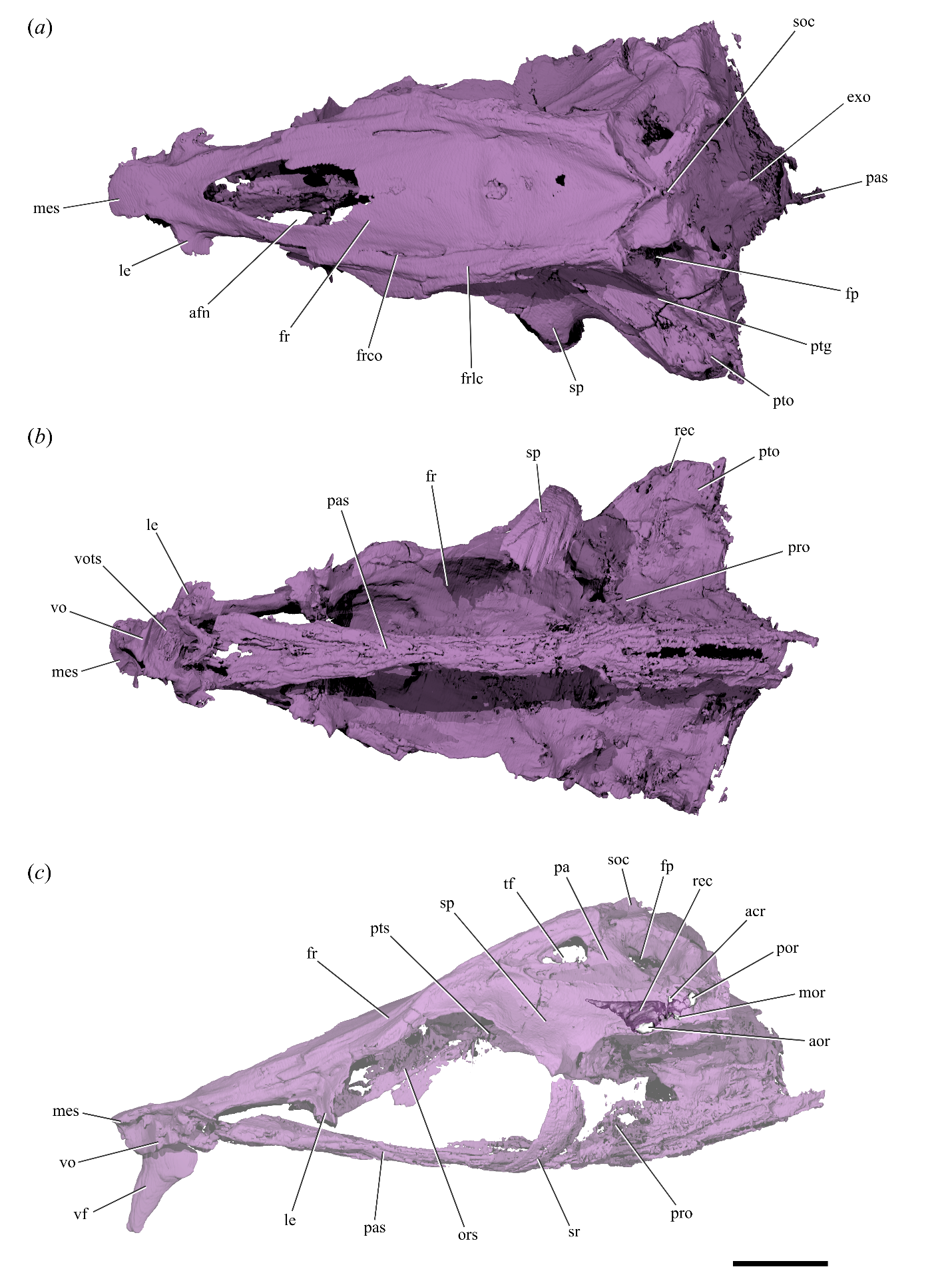


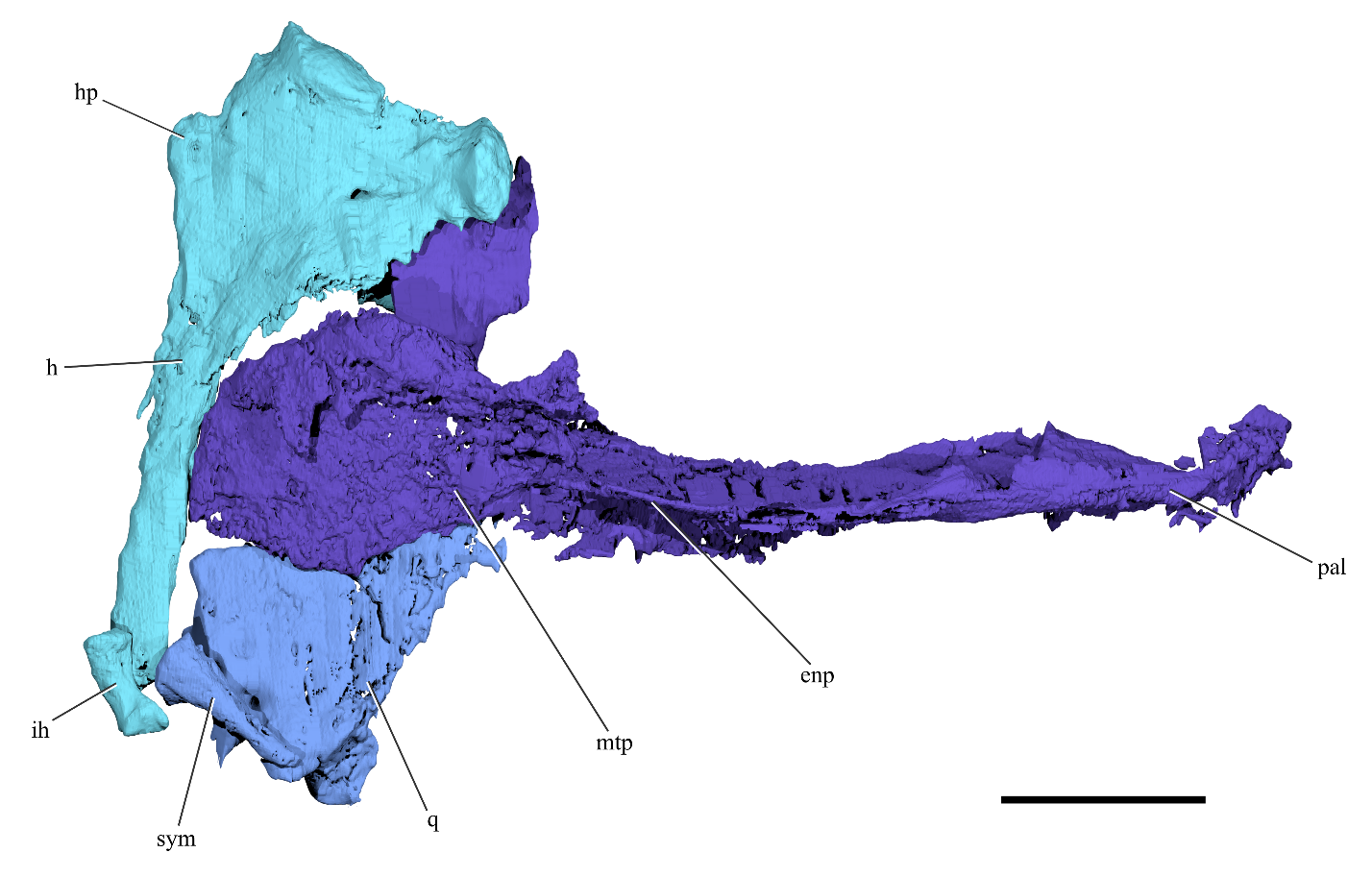


**Supplementary Figure S4.** Palate and suspensorium of †*Clupeopsis straeleni*. Rendering of left lateral palato-suspensorium of †*Clupeopsis* in medial view. Scale bar: 10 mm. Abbreviations: enp, endopterygoid; ih, interhyal; h, hyomandibula; hp, hyomandibular process; mtp, metapterygoid; pal, palatine; q, quadrate; sym, symplectic.


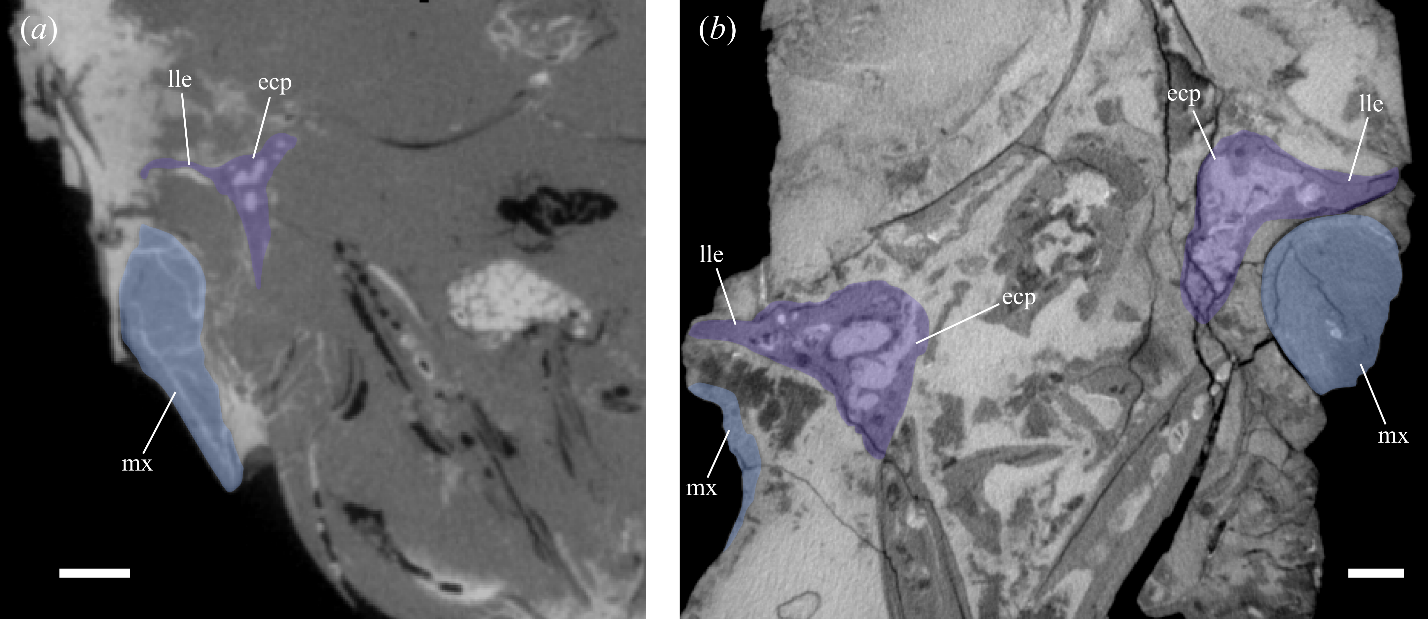


**Supplementary Figure S5.** Lateral horizontal lamina of the ectopterygoid in †*Clupeopsis* and †*Monosmilus*, as seen in cross-sectional tomograms. Transverse plane tomograms of (*a*) †*Clupeopsis straeleni* and (*b*) †*Monosmilus chureloides*, highlighting the ectopterygoid(s) in violet and the maxilla(e) in blue. Original tomograms have been mirrored to have left side of the specimens on the left and right side of the specimens on the right. Scale bars: 2 mm. Abbreviations: ecp, ectopterygoid; lle, lateral lamina of the ectopterygoid; mx, maxilla.


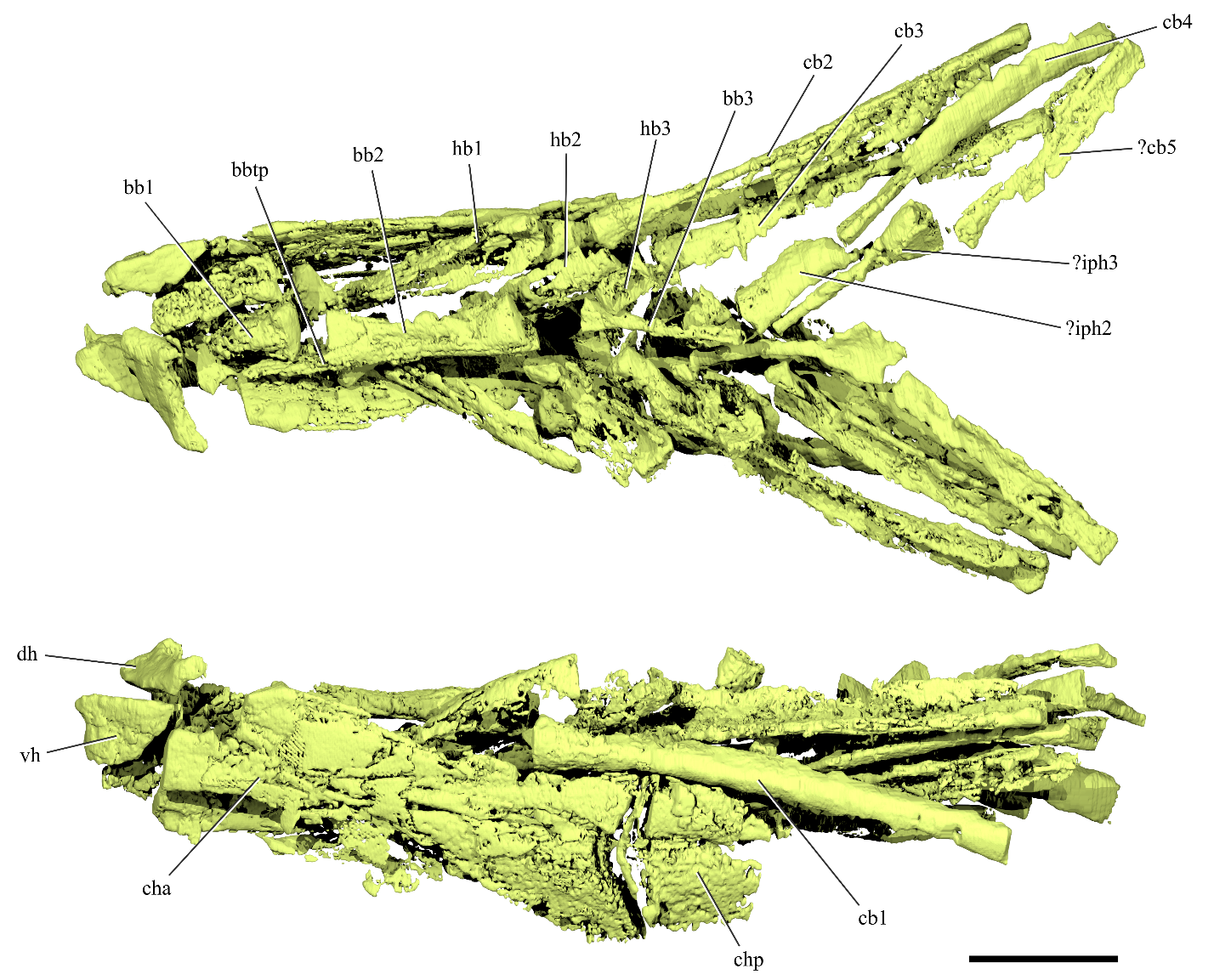


**Supplementary Figure S6.** Branchial skeleton of †*Clupeopsis straeleni*. Renderings of the gill skeleton of †*Clupeopsis* in dorsal (*top*) and left lateral (*bottom*) views. Elements whose identity is uncertain are labeled with a question mark followed by a tentative identification. Scale bar: 10 mm. Abbreviations: bb1, basibranchial 1; bb2, basibranchial 2; bb3, basibranchial 3; bbtp, basibranchial toothplate; cb1, ceratobranchial 1; cb2, ceratobranchial 2; cb3, ceratobranchial 3; cb4, ceratobranchial 4; cb5, ceratobranchial 5; cha, anterior ceratohyal; chp, posterior ceratohyal; dh, dorsal hypohyal; hb1, hypobranchial 1; hb2, hypobranchial 2; hb3, hypobranchial 3; iph2, infrapharyngobranchial 2; iph3, infrapharyngobranchial 3; vh, ventral hypohyal.

**
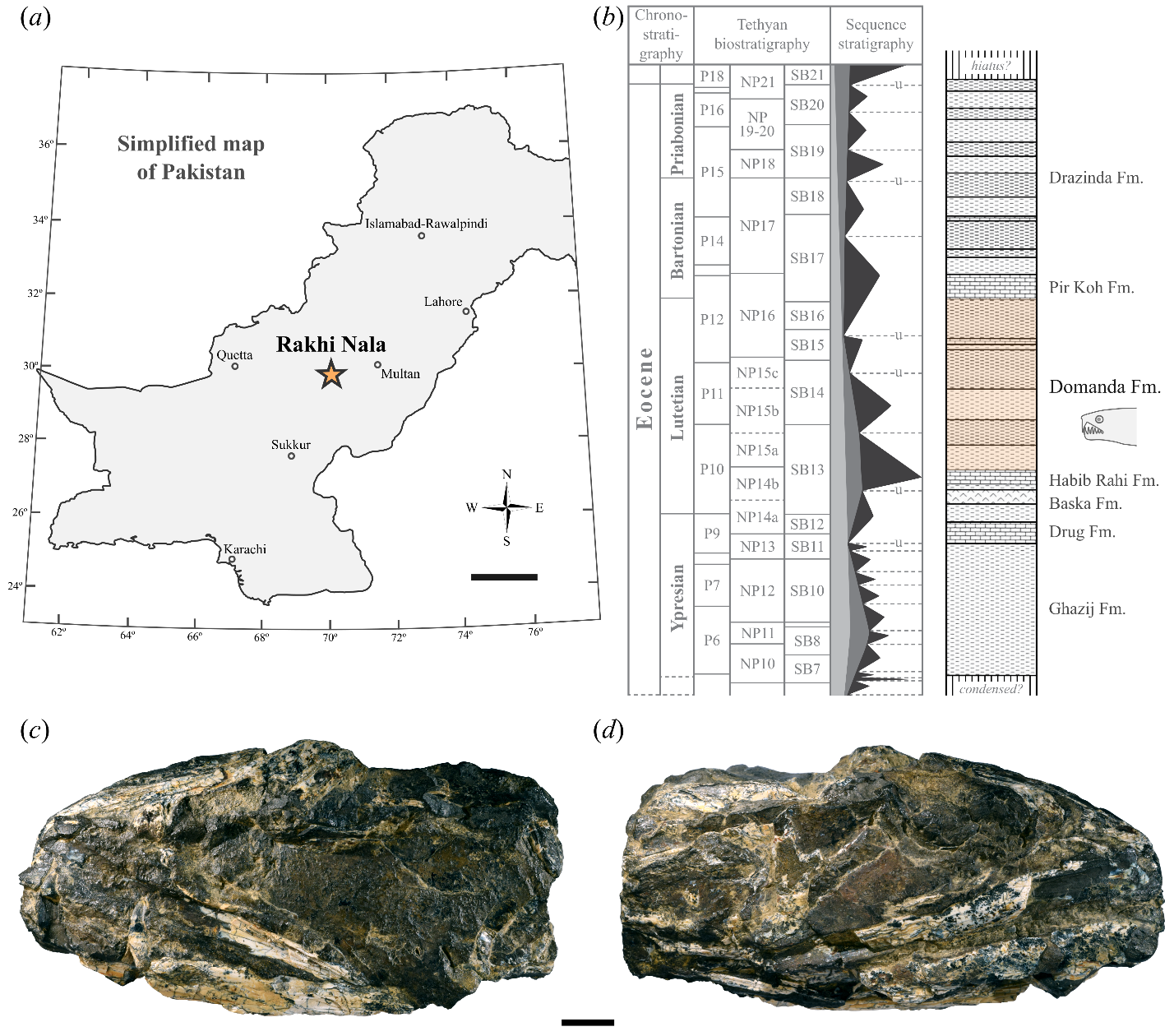
**

**Supplementary Figure S7.** †*Monosmilus chureloides* gen. et sp. nov., holotype (GSP-UM 37). (*a*) Simplified map of Pakistan showing the location of the Rakhi Nala locality, where the holotype of †*Monosmilus* has been found. Modified from ref. [13]. (*b*) Stratigraphic sequence of Eocene deposits exposed in Rakhi Nala, highlighting the Domanda Formation where the holotype of †*Monosmilus* has been found. For the sequence stratigraphy column, shaded regions represent relative sea level and ‘u’ indicates an unconformity. Modified from ref. [14]. Photographs of the †*Monosmilus chureloides* holotype in (*c*) left lateral and (*d*) right lateral views. Scale bars: (*a*), 200 km; (*c*), (*d*), 10 mm.


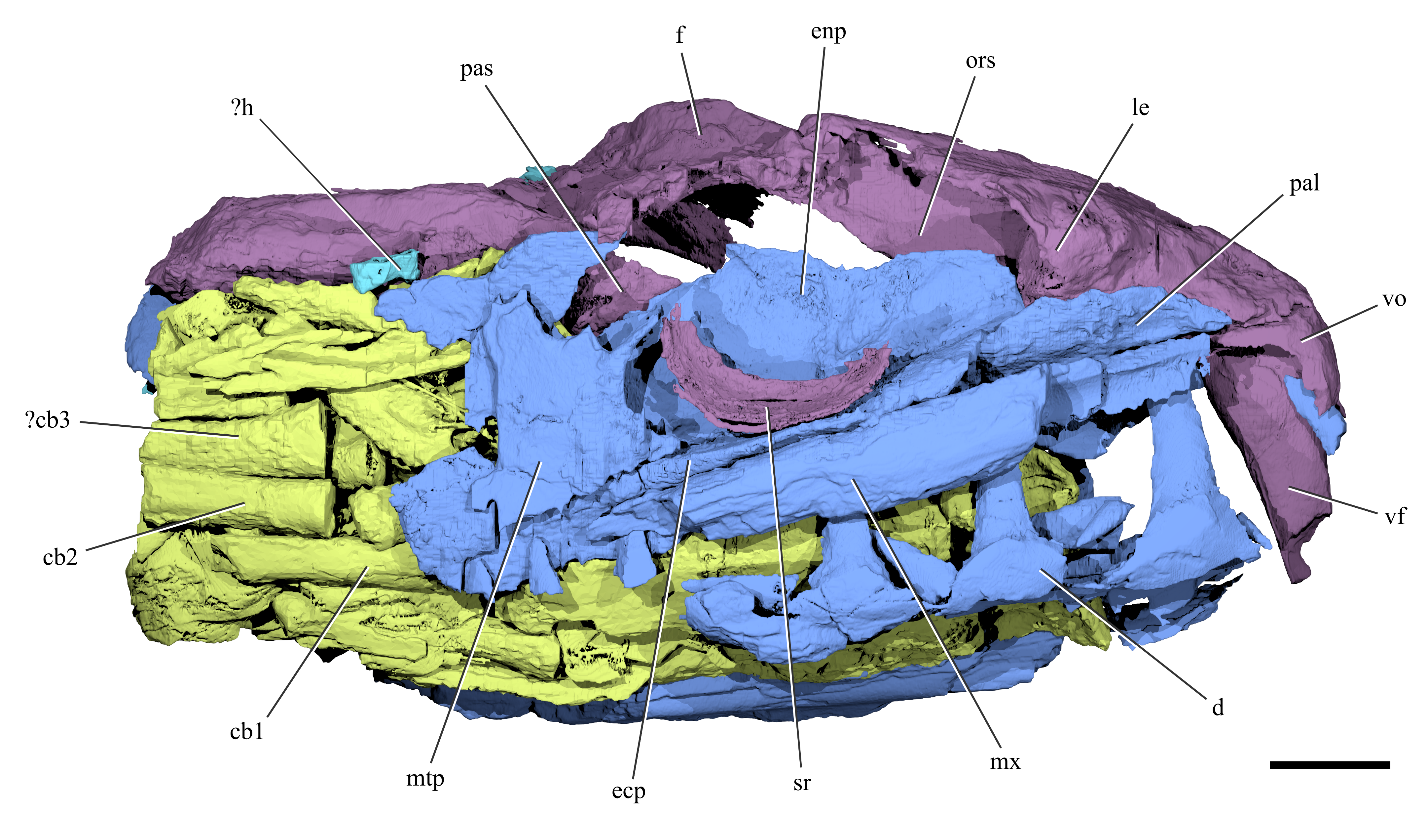


**Supplementary Figure S8.** Cranial anatomy of †*Monosmilus chureloides*. Rendering of †*Monosmilus* skull in right lateral view. Elements whose identity is uncertain are labeled with a question mark followed by a tentative identification. Colors indicate different cranial regions: neurocranium (purple), jaws, palatoquadrate and cheek bones (blue), dorsal portion of hyoid arch (cyan), branchial skeleton (chartreuse). Scale bar: 10 mm. Abbreviations: cb1, ceratobranchial 1; cb2, ceratobranchial 2; cb3, ceratobranchial 3; d, dentary; ecp, ectopterygoid; enp, endopterygoid; f, frontal; h, hyomandibula; le, lateral ethmoid; mtp, metapterygoid; mx, maxilla; pal, palatine; pas, parasphenoid; ors, orbitosphenoid; sr, scleral ring; vf, vomerine fang; vo, vomer.


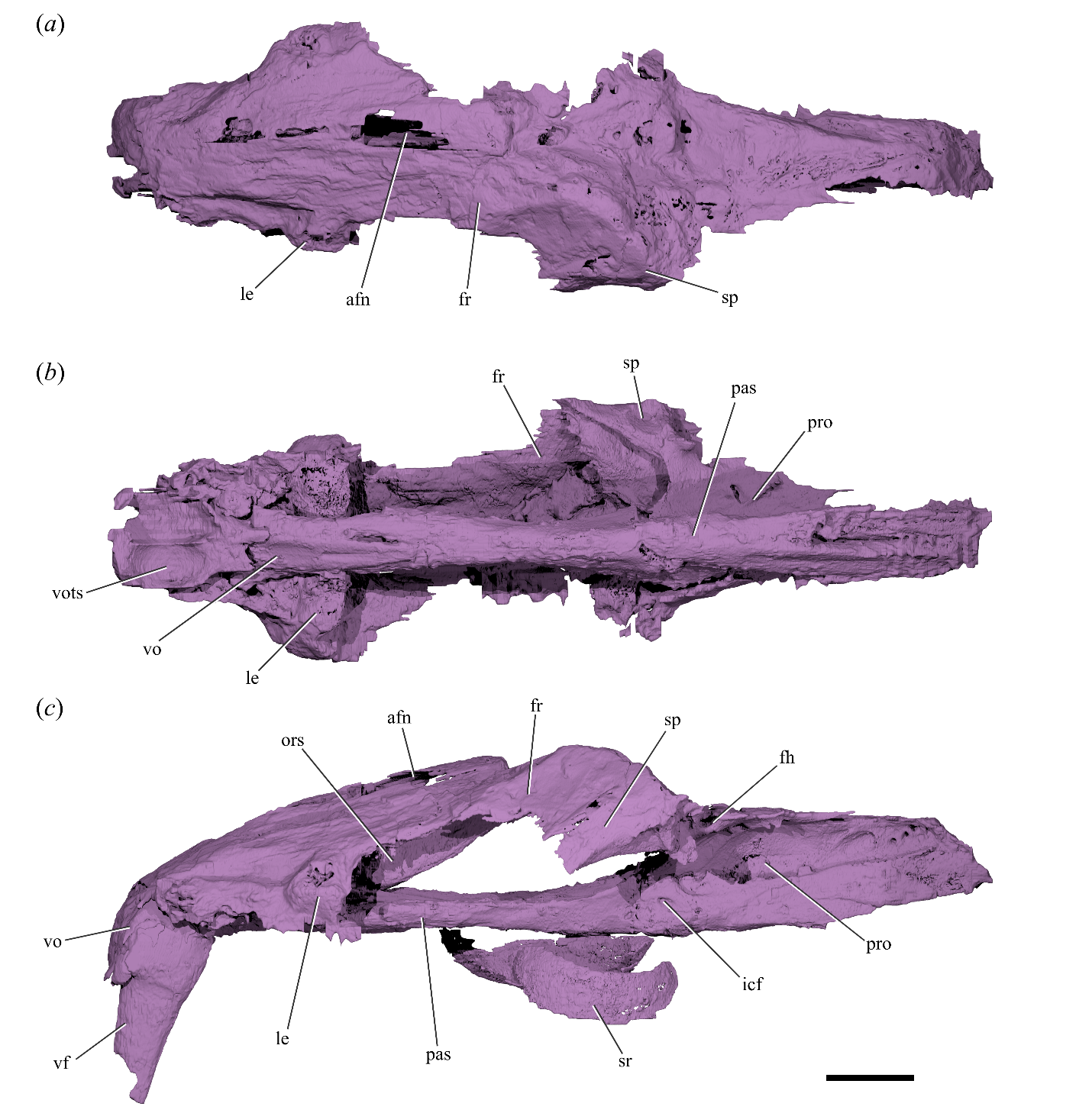


**Supplementary Figure S9.** Neurocranium of †*Monosmilus chureloides*. Renderings of †*Monosmilus* neurocranium in (*a*) dorsal, (*b*) ventral and (*c*) left lateral views. Scleral rings and vomerine fang not shown in (*a*) and (*b*) for clarity. Scale bar: 10 mm. Abbreviations: afn, anterior frontal fontanelle; fh, foramen for the hyomandibular trunk of cranial nerve VII; fr, frontal; icf, foramen in parasphenoid for internal carotid artery; le, lateral ethmoid; ors, orbitosphenoid; pas, parasphenoid; pro, prootic; sp, sphenotic; sr, scleral ring; vf, vomerine fang; vo, vomer; vots, vomerine tooth socket.

**
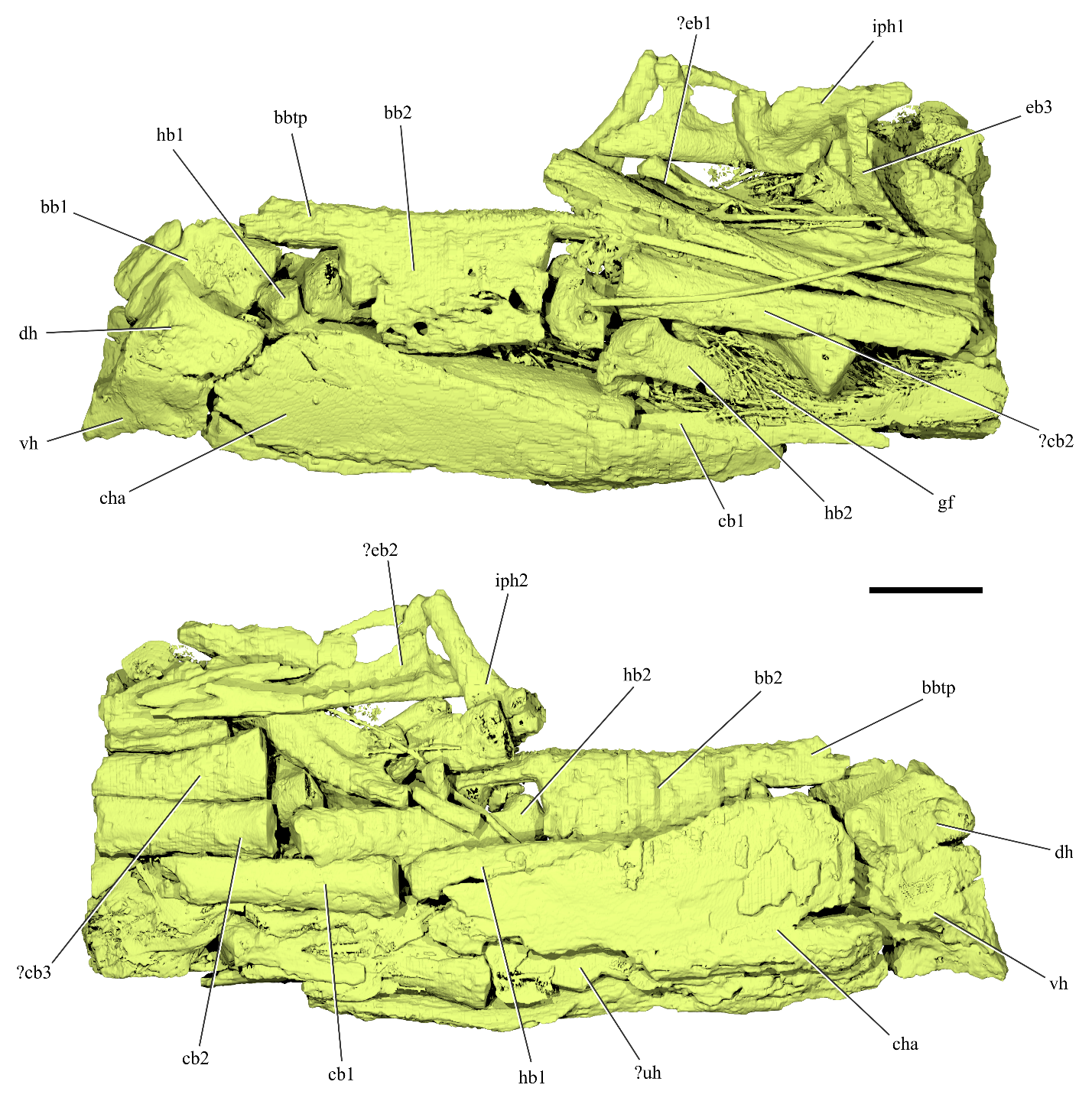
**

**Supplementary Figure S10.** Branchial skeleton of †*Monosmilus chureloides*. Renderings of the gill skeleton of †*Monosmilus* in left lateral (*top*) and right lateral (*bottom*) views. For paired bones, left-side elements are labeled only in left lateral view, while right-side elements are labeled only in right lateral view. Elements whose identity is uncertain are labeled with a question mark followed by a tentative identification. Scale bar: 10 mm. Abbreviations: bb1, basibranchial 1; bb2, basibranchial 2; bbtp, basibranchial toothplate; cb1, ceratobranchial 1; cb2, ceratobranchial 2; cb3, ceratobranchial 3; cha, anterior ceratohyal; dh, dorsal hypohyal; eb1, epibranchial 1; eb2, epibranchial 2; eb3, epibranchial 3; gf, mineralized gill filaments; hb1; hypobranchial 1; hb2, hypobranchial 2; iph1, infrapharyngobranchial 1; iph2, infrapharyngobranchial 2; vh, ventral hypohyal; uh, urohyal.


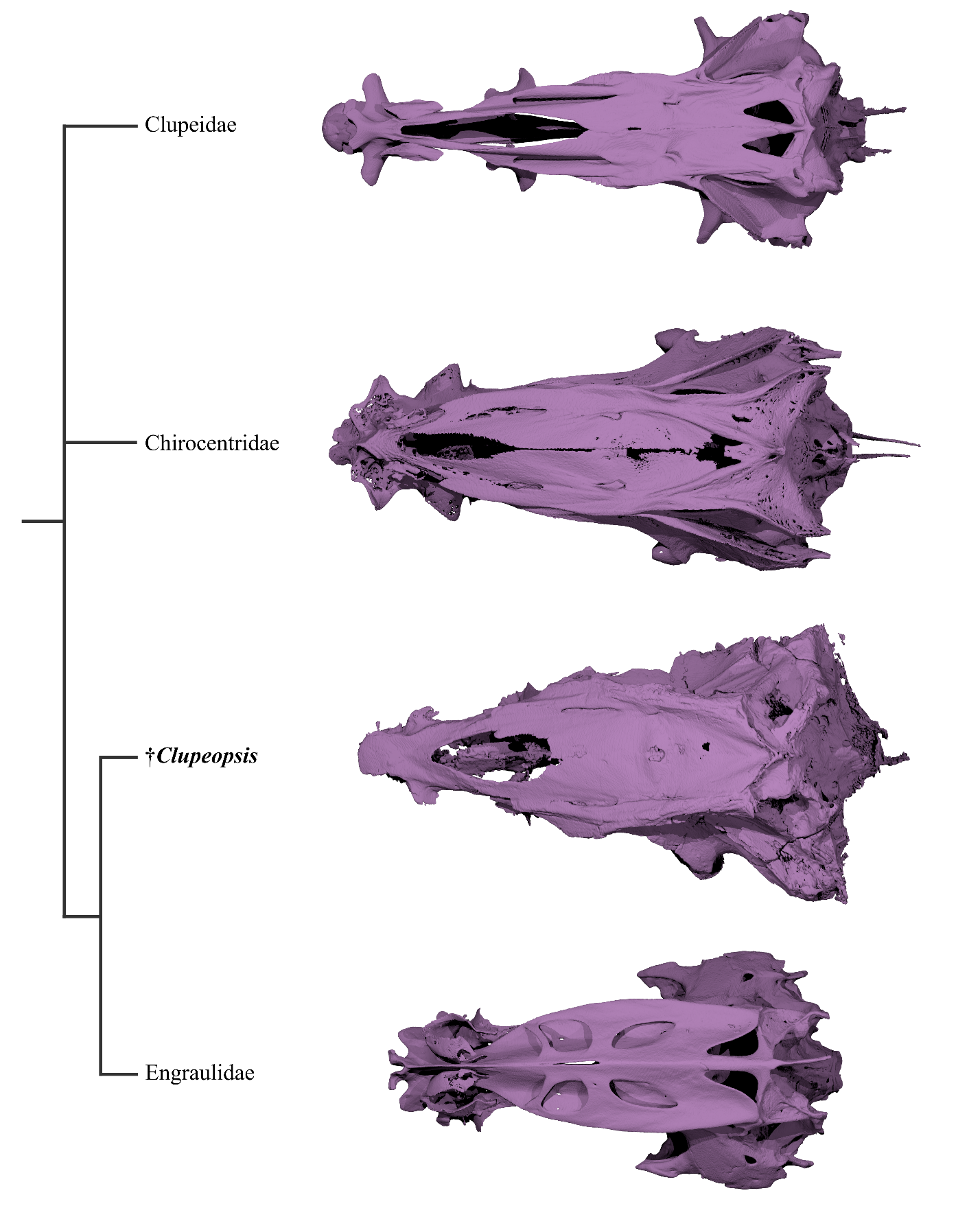


**Supplementary Figure S11.** Comparative morphology of the neurocranium in †*Clupeopsis* and other clupeiforms. Dorsal view of braincases belonging to representative clupeiforms. From top to bottom: *Odaxothrissa mento* (Clupeidae), *Chirocentrus dorab* (Chirocentridae), †*Clupeopsis straeleni* (total-group Engraulidae), *Setipinna* [*Lycothrissa*] *crocodilus* (Engraulidae). Renderings not at same scale.


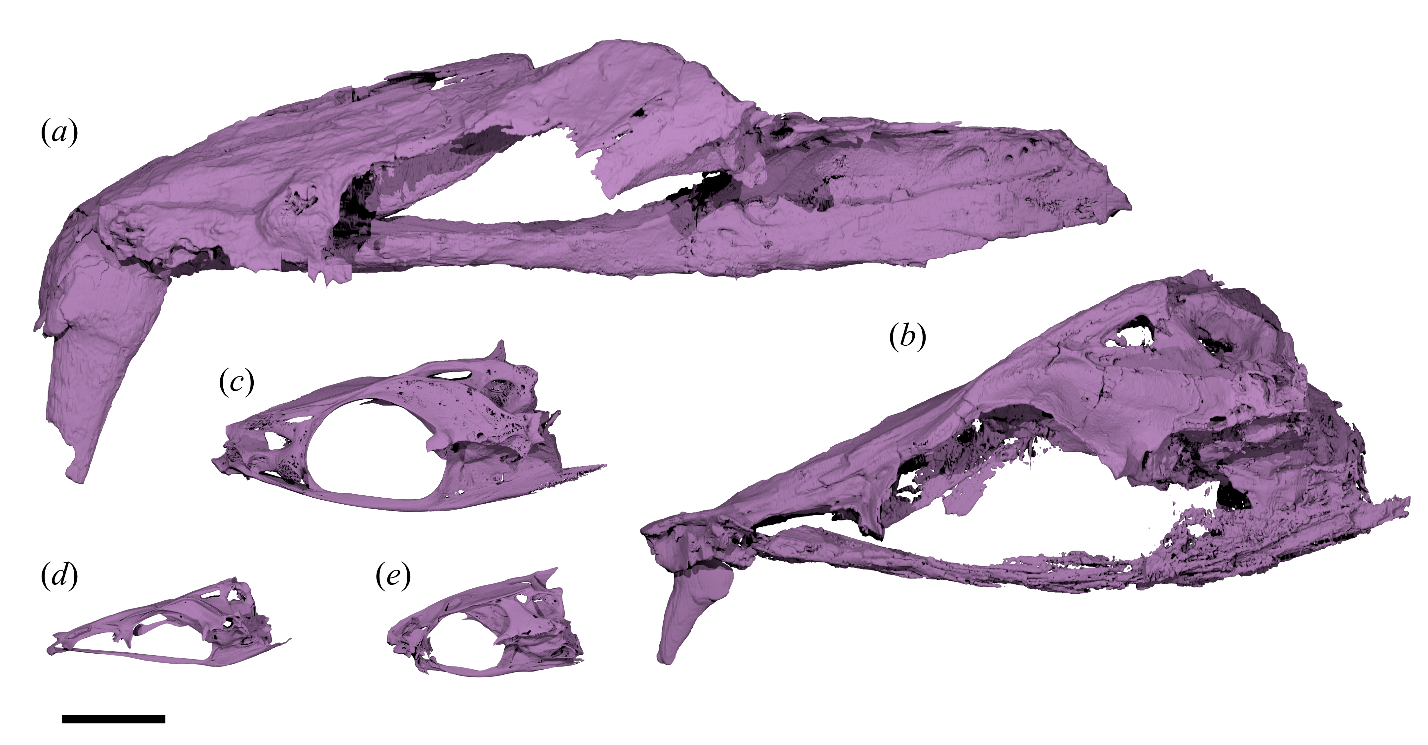


**Supplementary Figure S12.** Size comparison between sabre-toothed stem-anchovies and extant clupeiforms**.** Left lateral views of braincases belonging to the sabre-toothed stem Engraulidae, (*a*) †*Monosmilus chureloides* and (*b*) †*Clupeopsis straeleni*, and to representative extant clupeiforms, (*c*) *Chirocentrus dorab*, (*d*) *Odaxothrissa mento* and (*e*) *Setipinna* [*Lycothrissa*] *crocodilus*. Renderings at same scale. Scale bar: 10 mm.


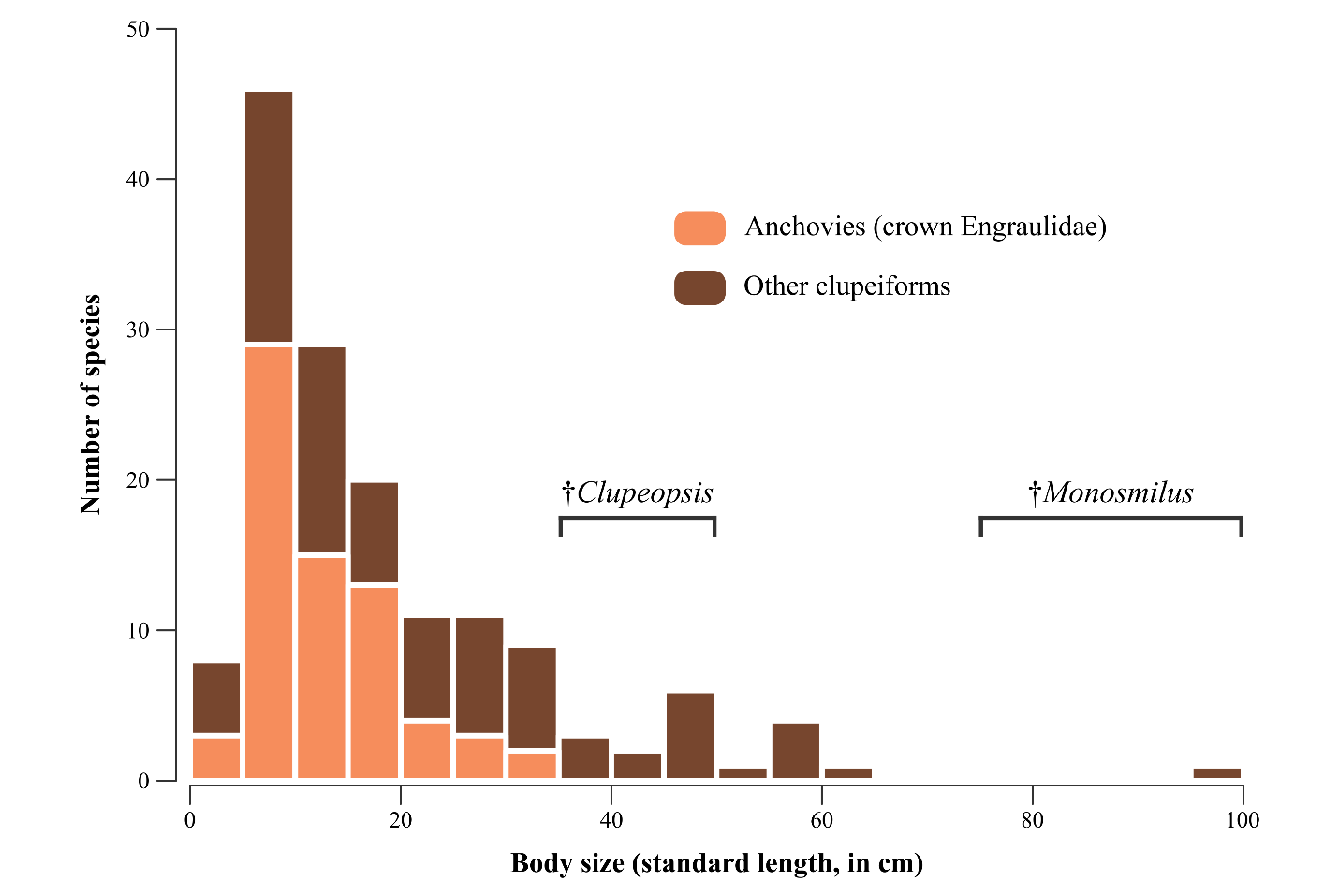


**Supplementary Figure S13.** Histogram displaying the distribution of body sizes in extant engraulid (orange) and other clupeiform (brown) species, based upon ref. [15]. Approximate estimates for †*Clupeopsis straeleni* and †*Monosmilus chureloides* were obtained by performing linear regressions of head length vs standard length in extant chirocentrid (upper estimate; *n* = 3, R^2^ = 0.9853) and engraulid (lower estimate; *n* = 37, R^2^ = 0.6232) specimens. Chirocentrids were chosen because they represent the upper limit of body sizes in extant clupeiforms and because †*Clupeopsis* likely had similar body proportions (based on what is preserved in the only known specimen for this taxon); engraulids were chosen because they are the closest living relatives of the two fossil taxa. The head length of †*Clupeopsis* was measured at 75 mm, while the head length of †*Monosmilus* was estimated at 150 mm (based on the incomplete skull representing the only known specimen for this taxon). Head length and standard length measurements for the linear regressions were taken from specimen photographs of chirocentrids and engraulids in FishBase (<https://www.fishbase.us/>).

**References**

[1] Delvaux, E. & Ortlieb, J. 1887 Les poisons fossiles de l’argile ypresienne de Belgique. – Description paléontologique accompagnée de documents stratigraphiques pour servir à l’étude monographique de cet étage. *Annales de la Société Géologique du Nord* **15**, 50-66.

[2] Casier, E. 1946 La faune ichthyologique de l'Yprésien de la Belgique. *Mémoires du Musée Royal d'Histoire Naturelle de Belgique* **104**.

[3] Steurbaut, E. & King, C. 2017 The composite Kortrijk section (W Belgium): a key reference for mid-Ypresian (Early Eocene) stratigraphy in the southern North Sea Basin. *Geologica Belgica* **20**, 125-160.

[4] De Coninck, J. 1986 Microfossiles à paroi organique de l’Yprésien inférieur à Quenast. *Professional Paper 1986, Ministère des Affaires éconimiques* **224**, 1-59.

[5] Debacker, T., Verniers, J., Piessens, K., Dewaele, S. & De Vos, W. 2015 Het onder-Paleozoïcum en het Massief van Brabant. In *Geologie van Vlaanderen* (ed. M. Borremans), pp. 18-56. Ghent, Academia Press Gent.

[6] Steurbaut, E. 1998 High-resolution holostratigraphy of middle Paleocene to early Eocene strata of Belgium and adjacent areas. *Palaeontographica Abteilung A* **247**, 91-156.

[7] Steurbaut, E., De Ceukelaire, M., Lanckacker, T., Matthijs, J., Stassen, P., Van Baelen, H. & Vandenberghe, N. 2016 An update of the lithostratigraphy of the Ieper Group. (

[8] Stassen, P., Speijer, R.P., Devleeschouwer, X., Abels, H., King, C., Willems, W. & Steurbaut, E. 2014 Eocene hyperthermals in the North Sea Basin: a Belgian Ypresian perspective. *Rendiconti Online della Società Geologica Italiana* **31**, 209-210.

[9] Westerhold, T., Röhl, U., Frederichs, T., Agnini, C., Raffi, I., Zachos, J.C. & Wilkens, R.H. 2017 Astronomical calibration of the Ypresian timescale:implications for seafloor spreading rates and the chaotic behavior of the solar system? . *Climate of the Past* **13**, 1129-1152.

[10] Dumont, A.H. 1850 Rapport sur la carte géologique du Royaume. *Bulletins de l’Académie Royale des Sciences, des Lettres et des Beaux-Arts de Belgique* **16**, 351–373.

[11] Leriche, M. 1092 Les Poissons paléocènes de la Belgique. *Mémoires du Musée Royal d’Histoire naturelle de Belgique* **2**, 1-67.

[12] Di Dario, F. 2004 Homology between the recessus lateralis and cephalic sensory canals, with the proposition of additional synapomorphies for the Clupeiformes and the Clupeoidei. *Zoological Journal of the Linnean Society* **141**, 257-270.

[13] Gingerich, P.D., Russell, D.E., Sigogneau-Russell, D., Hartenberger, J.-L., Shah, S.M.I., Hassan, M., Rose, K.D. & Ardrey, R.H. 1979 Reconnaissance survey and vertebrate paleontology of some Paleocene and Eocene formations in Pakistan. *Contributions from the Museum of Paleontology, University of Michigan* **25**, 105-116.

[14] Gingerich, P.D. 2003 Stratigraphic and micropaleontological constraints on the middle Eocene age of the mammal-bearing Kuldana Formation of Pakistan. *Journal of Vertebrate Paleontology* **23**, 643-651.

[15] Bloom, D.D., Burns, M.D. & Schriever, T.A. 2018 Evolution of body size and trophic position in migratory fishes: a phylogenetic comparative analysis of Clupeiformes (anchovies, herring, shad and allies). *Biological Journal of the Linnean Society* **125**, 302-314.
